# Supplementary material for: Who is (not) complying with the U. S. social distancing directive and why? Testing a general framework of compliance with virtual measures of social distancing
Source: PLoS One. 2021 Feb 24;16(2):e0247520. doi: 10.1371/journal.pone.0247520 (PMC7904183; doi:10.1371/journal.pone.0247520)
Supplement: S2 Material — (PDF) [file pone.0247520.s002.pdf]

S2 Material

Demographic Details for Each of the Four Sub-studies

As noted in the main text, all participants completed a core survey, but also were randomly assigned to four distinct surveys with specific questions. The four subsets involved: (a) source beliefs and science literacy, (b) news sources and belief in conspiracy theories, (c) compassion and concern for others vulnerability to COVID-19, and (d) perceived vulnerability to disease and disgust sensitivity. The following table presents demographic details for each of the four groups, as well as the total sample. As expected on the basis of the random assignment, the groups do not differ on any of the three variables (all *p*'s > .30).

Variable		Full Sample	Subset A	Subset B	Subset C	Subset D
N		2001	497	499	499	506
Gender						
	Female	903	229	224	214	236
	Male	1084	265	269	283	267
	Unreported	14	3	6	2	3
Age						
	Min-Max	18-89	18-89	18-77	19-76	18-76
	Mean	38.66	39.29	38.64	38.11	38.60
	SD	12.33	12.54	12.08	12.38	12.32
Political Orientation[a]						
	Mean	3.60	3.70	3.65	3.50	3.56
	SD	1.84	1.80	1.86	1.86	1.84

[a]Measured on a 1 "Extremely Liberal" to 7 "Extremely Conservative" scale.
