# Supplementary material for: Who is (not) complying with the U. S. social distancing directive and why? Testing a general framework of compliance with virtual measures of social distancing
Source: PLoS One. 2021 Feb 24;16(2):e0247520. doi: 10.1371/journal.pone.0247520 (PMC7904183; doi:10.1371/journal.pone.0247520)
Supplement: Attachment — Submitted filename: Response to Reviewers.docx [file pone.0247520.s003.docx]

**Response to Reviewers**

We would like to thank the editor and reviewers for providing such thoughtful and constructive feedback regarding our manuscript (“Who Is (Not) Complying with the Social Distancing Directive and Why?  Testing a General Framework of Compliance with Multiple Measures of Social Distancing,” PONE-D-20-30793). We were pleased to read the generally favorable responses to this work, and we are excited to have the opportunity to revise and resubmit this manuscript. We have made a number of changes in response to the reviewers’ feedback, and we feel that the resulting manuscript is much stronger as a result. We believe that these changes have addressed all comments and concerns.

In the pages that follow, we respond to each of the individual points made by the two reviewers. Each of the reviewers’ specific points is itself reproduced in this blue font and the responses in this black font.

**Reviewer 1**

This paper tests a theoretical model of compliance with social directives in the context of social distancing behaviors relevant to COVID-19 infection. The research is novel in testing a new model and comprehensive in testing multiple predictors of both self-reported and objectively measured behavior. The model is assessed using a cross-sectional survey of 2,000 MTurk workers. The data, analysis, and write-up are all of a high standard. Overall, I am very favorably disposed towards publication of this research in PLoS One.

We very much appreciate this encouraging assessment, as well as the thoughtful feedback provided in the comments below.

There are a small number of issues that the authors might wish to consider in revising the manuscript.
1. I found theoretical framework compelling and liked the division of predictors into beliefs about the context, beliefs about the source, and target characteristics. I can see the conceptual basis of the distinctions and wondered if this categorization can also be supported empirically (e.g., via factor or cluster analysis)?

This is a great suggestion, and one that we would certainly test if we were able. Unfortunately, though, the “planned missing” design that we employed prevents us from conducting such an analysis. That is, since participants completed only a subset of our measures, we are not able to enter all of our predictors into the model simultaneously, as would be necessary for the factor or cluster analyses you mention. We agree, though, that we would predict such a divergence, and this is something that we hope to test in future research. In the meantime, we have included this point in our discussion of limitations regarding the research on pp. 25-26 of the revised manuscript.

2. The behavioral measure of social distancing is very clever and adds an interesting dimension to the research. At the same time, is it accurate to suggest that it is an “objective” measure of “behavior”? The scenarios are hypothetical, and participants are asked about their comfort levels within each one. Is this more a measure of willingness (or something else) than behavior? If so, would it be worthwhile to predict discrepancies between willingness and self-reported behavior?

Thanks for these points. We agree that the virtual behavioral measure differs in important ways from real-life decision making, and we certainly do not wish to overstate what this measure can and cannot tell us about real-world behavior. In the revised manuscript, we have made several changes to provide some additional nuance and address this point. This includes more cautious wording in our description of our behavioral measure (e.g., we now use the term “virtual behavioral measure” instead of “behavioral measure” throughout, and we do not refer to this measure as offering an “objective” measure of behavior). Second, and more importantly, we have also added additional information regarding the predictive validity of these measures. In particular, we now discuss recent longitudinal data that were collected and analyzed subsequent to the submission of our original manuscript. The findings demonstrate that these same virtual measures prospectively predict who contracts COVID-19—and even *outpredict* self-report measures of social distancing (p. 7). We believe that these data provide compelling evidence that these virtual behavioral measures go beyond self-reports to tell us something important about how people are actually behaving in the real world.

Following your suggestion, we also have added a new section to the paper in which we predict discrepancies between our self-report and virtual behavioral measures (pp. 22-24). We believe that this section provides some interesting additional information regarding the nature of our two distancing measures, as well as the nature of the relations between our various predictors and social distancing.

3. The differences between the factors that predict “objective” and self-report measures of behavior are intriguing. Could the analyses go further and determine whether these seeming differences are significant? For instance, could MLM be used here with the two outcomes as a within-participants factor?

The revised manuscript now includes a section that reports significance tests statistically comparing the correlations of the predictor variables with the virtual behavior measure to those with the self-report measure. Table 3 lists all variables for which the correlations differed to a statistically significant degree. Of major interest is that test of COVID-19 knowledge – one of the most objective measures employed in the study – correlates substantially higher with the virtual behavior measure than the self-report measure of social distancing. In contrast, a variable that is among the most subjective – expressed support or opposition to the social distancing directive – correlates substantially more strongly to the self-report measure.

4. Table 1 offers a long list of significant predictors of social distancing (attesting to the model’s value) and I appreciated the multivariable analyses of predictors within each set of beliefs. Would the research benefit from an additional analysis that included all 19 beliefs in the model to determine which beliefs or characteristics are most important, and how much variance in the outcomes they collectively explain?

We agree that this had the potential to be an informative analysis. However, as noted above, our planned missing design prevents us from conducting such an analysis. Again, this cost of our strategic design decision is discussed as a limitation in the Discussion section (p. 25).

**Reviewer 2**

The authors present an article that reports upon the findings of an online, survey-based study designed to identify more about the antecedents of people’s willingness to comply with social distancing measures in the wake of the COVID-19 pandemic. Two distinct DVs are used; self-report measures and a novel ‘behavioral’ measure.

The research is timely, given the unfolding nature of the global pandemic, and there are also potential theoretical and applied implications for the work. However, there are a number of significant issues with the study – as reported – that I feel preclude publication on PLOS One at this time.

A key issue is the lack of detail provided in each of the sections of the study (particularly the introduction, methods and discussion). There needed to be more methodological detail to allow for the replication of this study, if necessary. In terms of the results, I think that the basic analysis is okay, but I wonder whether the authors could be more ambitious with their modelling. The discussion is also notably light on detail – more effort needed to be made to couch the findings of the study among those from the rich, extant literature on science communication.

Thanks for these points. We appreciate the favorable reactions concerning the timely nature of the research and the potential theoretical and applied implications of the findings. Regarding the methods, we regret that our “planned missing” design was not clear in our initial manuscript and that the methodological presentation was not sufficiently detailed. We now realize that we had relied too heavily on the provision of all the measures in the OSF page for which we had presented a link. Instead of forcing readers to refer to the OSF information, we have made several additions to this revised manuscript to address this issue and ensure that we fully discuss all of the critical components of the study design, the measures, and the analysis details. These additions, along with the OSF page, should be useful to any researchers who may wish to replicate or extend our results.

It is possible that with major corrections/additions that this article can be shaped into a publishable offering. What follows is a specific appraisal of the key issues with the article, from my perspective. I wish the authors all the best in making the suggested amendments and will look forward to appraising the article in due course.

We appreciate the obvious time and effort that you put into reviewing the manuscript. We hope that the revised manuscript has satisfactorily addressed your comments and concerns. We believe that the manuscript has been strengthened considerably as a result of our attending your helpful comments.

Title

It should be stated within the title that the focus is on compliance within the US. The study is clearly tied to social distancing directives in the US and the study is conducted on a US sample. The current title infers that the scope of the study transcends the US context, which is not the case.

We agree that societal context is important here, and we certainly do not wish to overgeneralize—nor to lead readers to do so. We have changed the title so that we now explicitly specify that this research is focused on the US context.

Introduction.

The introduction would benefit from a short paragraph outlining the COVID-19 crisis and stating more about the situation and response in the US. Again, this is important to clarify the context and reach of the study, which is US-centric. This US-centricity is exemplified by the claim that “stay six feet away from others” is a regularly repeated mantra. This might be the case for the US, but this is not the case in Europe, for example, where the mantra has been more ‘metric’ (2 meters or 1 meter with extra precautions).

Following your suggestions, we have added additional detail about the pandemic and the US sociopolitical context throughout the manuscript. As examples, the opening paragraph has been modified, as has our presentation regarding how President Trump and state governors differed in their public statements during the early months of the pandemic in the US (p. 8).

Line 35 – I feel that the claim that people will only respond to directives that are justified needs to be more nuanced. There are examples of where people comply with directives that are not deemed to be well-justified, because they do not wish to suffer social/group disapproval.

Yes, the need for a more nuanced consideration of this issue is now clear. We have added a paragraph to the introduction in which we summarize Kelman’s classic distinction between passive conformity in the interest of approval and internalized, private acceptance of the inherent value of a message (p. 3). With this context, we are able to present our operational definition of compliance as engaging in the requested behavior for whatever reason. We also explicitly note a directive can be deemed justified either on the basis of mere respect for the source or on the basis of reasoning regarding the merits of the source’s arguments (p. 4), thus clarifying what we mean by the statement you highlighted.

Line 41 – Please outline a relevant (e.g. health-related) example of a study that evidences the point you are making here.

We have made such an addition in the revised draft. We now discuss research on conspiracy theory endorsement and parental vaccination decisions, which provides a useful illustration of these processes.

Line 44 – You have evidenced the source and audience as being important, but what about message factors? If you are treating message factors as part of the ‘surrounding context’ then I feel that this needs to be explicitly stated. If you are not classing message factors as important, then this needs to be strongly justified, dismissing the ‘what’ from the ‘who is saying what to whom?’ mantra would otherwise appear to be a significant oversight.

Thank you for the suggestion. A discussion of the Yale Communication Group’s classic persuasion framework concerning “who says what to whom?” now appears on p. 5. This context provided us with the opportunity to discuss manners in which the persuasion question and our compliance framework are both similar and different. Related to your specific concern, we explicitly note that our focus centers on how individuals respond to the general plea for behavioral change, not any specific persuasive message in service of that plea.

Line 49-50 – I think that the phrasing here needs to be more nuanced. It is not always going to be the case that people who process information in a favorable way will engage in the correct/sustained/sufficient behaviors (we see this a lot with health and environmental actions, where people often engage in compensatory behavioral trade-offs). Equally, sometimes people who are less positive about the directions of a message, might still comply with them.

We have re-worded the sentence so as to emphasize the probabilistic nature of the statement.

Line 58 – Please provide a reasoned example of the characteristics/beliefs that might affect mis-representation of social distancing on self-report measures.

Two such examples are now offered on pp. 5-6. We consider the potential biasing influence of an individual’s political ideology, as well as self-beliefs that one is a compassionate person. They nicely illustrate the argument.

Line 66 – I need more convincing that the novel behavioral measures you have designed have the ecological validity that you are claiming. Granted they do present people with in the moment behavioral decisions, but these are virtual and vicarious. Please provide a fuller justification of the validity of your ‘graphical scenarios’ in emulating real-life decision making.

We understand this concern, and we have added a fuller justification of the validity of these measures in our revised draft. This includes both a deeper discussion of the representativeness of the virtual situations themselves, and, more importantly, a discussion of recent longitudinal data showing that these same virtual measures prospectively predict who contracts COVID-19, as noted in our response to Reviewer 1’s point #2 above. Together, we feel that these additions offer compelling support for the value of the virtual behavior measures.

Line 75-112 – There needs to be a fuller, theoretical justification for the selection of the various predictors used in this study (source, context and target). While I am not disputing the relevance of the predictors you have selected, the selection process is opaque, and the selection of items appears subjective and unsystematic. For example, your decision to look at ‘objective knowledge of COVID-19’ as opposed to ‘subjective knowledge’ is interesting, given that subjective knowledge of science/technological issues often shares a stronger relationship with acceptance (see, e.g., some of the work by Stugis and Allum).

Tying the selection of variable more explicitly to an established over-arching theoretical framework(s) would have made for a more compelling narrative. It would have also allowed you to be more adventurous in modeling moderation/mediation pathways.

We regret that the theoretical justification for our predictor variables was not clear in the initial draft. In our revised manuscript we have added additional detail throughout (e.g., p. 7). In particular, we emphasize that the selection of predictor variables was guided by our framework regarding the essential components of a directive.

Line 88 – You need to provide evidence for the claim that Trump downplayed the severity relative to state Governors (I believe you, but you need evidence).

We have added citations supporting this argument in the revised draft (p. 8).

Methods

Line 123 – Strictly speaking ‘MTurkers’ is not a word and so you should probably put this is ‘inverted commas’

We have removed the (non-)word “MTurkers” from the draft and have changed the wording to “MTurk workers” throughout.

Line 127 – I understand the arguments for creating four versions of the survey, but you should probably raise the fact that participants did not all complete the same survey as a limitation to the study within the discussion.

We agree, and we have added a consideration of this limitation to our Discussion section in the revised manuscript (pp. 25-26), as noted above in our response to Reviewer 1’s point #1 above.

Line 131 – Bearing in mind you have a between-subjects design, you need to show evidence that there has been successful randomization to condition in terms of core participant demographics. Please provide some evidence of this – means/frequencies, plus basic statistical comparative analysis will be sufficient.

We have added this information to the Supplemental Material, as we now note on page 10.

Line 136 – Related to my point above, you need to have specified that there was a national lockdown in the US (and the nature of this lockdown), rather than assuming that the readership of the article will know/be aware of this.

We have added this specific information, as well as a deeper discussion of the societal context in the US to our revised manuscript (e.g., pp. 2-3).

Line 140 – Please specifically refer to COVID-19 as there could be other COVIDs in the future (although we hope not of course!)

That’s a good (although unnerving) point. We’ve made this change.

Line 144 – What kind of selection, piloting and pre-testing did your social distancing behavioral measures go through? Now, they might have a degree of face-validity as measures of social-distancing compliance, but what formal evidence is there of their content-validity beyond this? While I do applaud the innovation in measurement here, I am concerned that that drawing strong inferences based on an un-proven, un-tested measure is ill-advised. As such you need to provide a fuller account of the selection and development of these measures, while also acknowledging the limitations around their uses within the discussion.

Thanks for this point. We have addressed this in the revised manuscript. Most importantly, as noted in our response to Reviewer 1’s point #2 above, we have included additional discussion and evidence regarding the validity of these measures—and, in particular, their predictive validity with respect to who contracts COVID-19.

Line 156-233 – You need to include the responses options for all your measures. You also need to provide access (probably in the form of an appendix or supplementary information) to your questionnaire measures, so people can see all the questions that were asked and how they were asked. Within the text you might wish to add two example questions/statements per scale outlined.

Your comment made it apparent that we had relied too heavily on our having made the full set of measures available on the OSF site for this project, as noted on p. 11 of the current version. We agree that this will be helpful information for readers, and we have included these additional details (example scale items and response options) in the revised manuscript.

Line 160 – Did you assess people’s awareness and understanding of the social distancing recommendations? If not, this is a limitation that needs consideration within the discussion.

We did not assess awareness/understanding, although we agree that this would have been a valuable measure to include. Our assessment was that the high-intensity messaging environment, combined with the relatively simple nature of the social-distancing regulation (in the US, “stay 6 feet away from others”) should have led to relatively full understanding across the board. However, our data cannot directly speak to that question, and we agree that this is a limitation. We have included mention of this in the paragraph concerning limitations that we added to the Discussion section (p. 26).

Line 168 – How many statements were used to assess COVID-19 knowledge?

There were 13 statements in total. We have noted that in the revised manuscript (p. 12).

Line 199 – For those outside the US it would help to know what political leaning each of these news/media sources have.

Excellent point. We have added this information here, along with the relevant citation (p. 14).

Line 202 – Revisit this sentence and see if you can rephrase it to make it clearer.

Yes, we clarified the sentence (p. 14).

Line 226 – What demographics were included and how were they assessed?

The demographic factors we assessed included age, gender, and employment status, as well as political orientation. We have added this information to the revised manuscript (p. 15).

Results

The results appear to be okay but very basic given the scope of the study and the data you have to work with. I wonder if you could have been more ambitious with your modeling/analysis, to say something a little more about who is responding to COVID-19 guidance or not?

This point of critique maps back to a point I made early re: the selection of the predictors, which – although not disputing their relevance - appears quite haphazard. Your narrative would be substantially more compelling if you were able to chart, model and report some moderation/mediation pathways. You have a considerable number of predictors here, which should make it possible, provided that theoretically justified pathways can be created.

We regret that the theoretical justification for our measures was not clear in our initial submission. As noted in our response to an earlier comment (that concerning Lines 75-112), we have added additional information regarding the theoretical framework/background for our various predictor variables in our revised manuscript. Once again, the specific predictor variables were inspired by our framework regarding the essential elements of a directive. We hope that this has addressed this issue.

Regarding the addition of moderation and/or mediation analyses: our planned missing design, as noted above, unfortunately does not allow for a full exploration of these questions. Because of this design feature, and because these analyses would be post-hoc/exploratory, and beyond the scope of the present paper, we have not pursued any moderation/mediation analyses. However, as noted in response to Reviewer 1’s points #2 and #3, the results section has been expanded with the inclusion of more extensive analyses concerning the comparison of the virtual behavior and self-report measures of social distancing (pp. 20-24).

Line 308 – Please state ‘COVID-19’.

Done.

Discussion

The discussion needs a lot of work. Lines 328-351 essentially just re-report detail that has just been outlined within the results section. As such, this detail can be pared back somewhat.

The discussion from Line 352 onwards is basic fails to map clearly and robustly to the rich literature that exists around science communication. The claims that are being made, need to be more couched within the extant literature and explicitly linked back to the study findings. There also needs to be a more upfront consideration of the limitations of the research.

The Discussion section has been revised considerably. In addition to the consideration of the limitations of the research that was noted above, we have modified our discussion of the implications of the research findings for public service campaigns encouraging social distancing. That discussion is now embedded within a context offered by the literature on science communication. We briefly review a few key points from this communication literature, highlighting in particular the difficulties that arise when a scientific issue becomes politicized. We then note that, although our findings are silent with respect to the strategies that might be optimal when attempting to promote social distancing, they do offer some insights regarding the content that should be emphasized.

*********

Once again, we wish to express our gratitude for the insightful comments offered by the reviewers. We certainly believe that our addressing the points that were raised has resulted in a stronger manuscript. We hope that you agree. Thank you for the opportunity to submit a revised manuscript for your consideration.

Sincerely,

Russell H. Fazio

Harold E. Burtt Chair in Psychology
