# Supplementary material for: Who is (not) complying with the U. S. social distancing directive and why? Testing a general framework of compliance with virtual measures of social distancing
Source: PLoS One. 2021 Feb 24;16(2):e0247520. doi: 10.1371/journal.pone.0247520 (PMC7904183; doi:10.1371/journal.pone.0247520)
Supplement: Attachment — Submitted filename: Response to Reviewers.docx [file pone.0247520.s004.docx]

**Response to Reviewers**

We would like to thank the editor and reviewers for their favorable reactions to our revised manuscript, PONE-D-20-30793_R1. To briefly summarize the responses, Reviewer 1 noted that the revision had been “very responsive to my suggestions and the changes to the manuscript made a very good paper even better.” He was “very happy” to recommend publication. Reviewer 2 also felt that the “article is now much improved” and viewed the revision as a thorough response to the previous comments. Reviewer 2 also offered “a few additional minor points” for us to consider. We have done exactly that with respect to each and every point, and we thank the reviewer for having spent so much time examining the manuscript. In almost all the cases, we made some revision in response to the reviewer’s remarks. In the few cases where we opted not to revise (specifically, the first two points below concerning altering the title and inserting an image of real-world social distancing measures), we explain our rationale.

In the pages that follow, we respond to each of the individual “minor points” offered by Reviewer 2. Each of the reviewer’s specific points is itself reproduced in this blue font and the responses in this black font.

Title, Abstract and Introduction

The title, abstract and introduction are now much improved, however, I have a couple of final suggestions for these sections:

1. I feel that the title would benefit from mentioning COVID-19 – as the work focuses on compliance with social distancing requests in response to this specific pandemic.

We appreciate this comment, which led us to re-visit our title once again. In response to the reviewer’s earlier suggestion, we had inserted “U.S.” into the title so as to clarify that the research was specific to that nation. Working “COVID-19” into what already is a long and relatively complex title strikes us as less critical, and we feel that the added complexity would make the title somewhat harder to read and understand. Moreover, the term “social distancing” arose specifically in response to the COVID-19 pandemic and, hence should readily cue this specific pandemic for anyone perusing the title. It therefore does not seem necessary to note that explicitly in the title. However, upon re-visiting title, we did decide to make a slight change. We substituted “virtual” for “multiple” measures of social distancing. So, the full title is now: “Who is (not) complying with the U. S. social distancing directive and why? Testing a general framework of compliance with virtual measures of social distancing.” We thought it best to highlight the “virtual measures” in the title because the findings so strongly feature the unique contributions offered by the virtual behavioral measure of self-distancing. Indeed, this seems all the more important in light of the longitudinal data that we summarize on p. 7 validating the virtual measure as a predictor of individuals’ likelihood of contracting COVID-19. In the earlier version of our manuscript, we had cited a preprint reporting this longitudinal finding. We now have updated the citation, as that paper has since been accepted, fast-tracked, and published in the *Proceedings of the National Academy of Sciences of the United States of America*.

On page 2-3, it might be beneficial to incorporate a couple of photographs to depict what the social measures (e.g. tape on the floor) look like. This should hopefully be simple to get hold of as University campuses tend to have such measures (if they are accessible during the pandemic)

Thanks for this suggestion. However, we currently have only very restricted access to our University campus. Nevertheless, such images are readily available on the internet. If we were writing for the popular press, we would eagerly include such photos, but the images are so common that their inclusion seems unnecessary in a scientific article.

Methods

The methods section is now fuller. Thank you for including more of the detail, rather than placing this all in the supplementary material. It might add to the article if you were to include a couple of ‘stills’ depicting the virtual behaviour measures. I understand these are in the ‘Supplementary Material’ but having a couple of images in the main text would be good for to illustrate the innovative method you have used.

Yes, this is an excellent idea. We now have included a figure that presents still images of three of the ten virtual behavior items (p. 11). The text also continues to encourage readers to visit our demonstration website, where all ten are displayed in the very same format in which they were presented to the participants in our study.

Results

Looking at Table 1, I am wondering how you coded the people who did not enter their gender? It could be that these individuals wished to withhold information about their gender, but equally it could be that these individuals identify as non-binary. In the spirit of inclusivity, you should probably add a footnote to Table 1 to note that people withholding gender were not included.

We have added a new footnote to the table that now offers this detail.

Overall, while I find the results section relatively simple bearing in mind the data-set that has been accrued, I sense that my requirement for more in-depth inferential analysis should be traded-off against the value of getting this article into the public realm (also, I appreciate the issues caused by the methods used). I would encourage you to perhaps outline in the discussion section what the obvious ‘next steps’ might be for the work, which might allow for more in-depth inferential statistics to be performed – one or two suggestions would suffice.

We added a sentence to the discussion section in which we expand upon this point.

I am wondering whether using a basic t-test to compare the strengths of the correlation coefficients is appropriate (see Table 3). This is something that you should check, as I feel that there might be more statistically robust ways of drawing such comparisons. For example, this recent article in PLOS offers up suggestions and techniques for drawing such comparisons: cocor: A Comprehensive Solution for the Statistical Comparison of Correlations (plos.org). If the current analysis is appropriate, then including a couple of references to studies that have used this method of analysis should provide adequate justification for the approach.

The statistical test that we employed to compare dependent correlations is very standard. Numerous citations can be provided to support the approach that we used. As you suggested, we have inserted one such citation.

P22 - you suggest that “Any predictor variable for which the comparison yielded a significance level greater than .05 is listed”; do you mean ‘lower than .05’?

Thank you for noticing this ambiguity. The sentence now reads: Any predictor variable for which the comparison yielded a *p*-value less than .05 is listed.

Also, on P22 you state “…it is interesting to consider how the two variables differ with respect to the unique variance for which they each accounted in the multiple regressions”. The end of this sentence suggests you have done a multiple (linear) regression analysis, when in reality you have done a multitude of regressions (unless I am mistaken). As such, you should probably look to reword this sentence.

Yes, we’ve re-worded, so as to now refer to “the unique variance for which they each accounted in the various multiple regressions reported in Table 1.”

P23 - you suggest that the skew in the self-report measure was due to people wishing to believe that they had acted in a way to lessen their and others’ risk. It could also be that people wished to present themselves in such a way. This should probably be accounted for in your statement.

We agree that this self-presentation explanation is a possibility, and we thought that our statement implied this possibility. However, we have revised the text so as to make this more explicit.

P25 - I find the paragraph beneath the table quite difficult to parse. Is there are way to rephrase things slightly to make it easier to understand.

Yes, thank you for bringing this to our attention. It is indeed difficult to communicate relations involving residuals. Hence, we have expanded the text a bit so as to clarify the meaning.

Discussion

P27 – do you have countries that you can reference where there was less politicisation of the pandemic? You might wish to mention South Korea, which seems to have done this effectively. Also, there are interesting contemporary articles showing how politicisation has affected the COVID response that you might wish to mention (e.g. Crayne and Medeiros, 2020). These articles might also be applicable to some of your commentary on politicisation on P28.

Crayne, M. P., & Medeiros, K. E. (2020). Making sense of crisis: Charismatic, ideological, and pragmatic leadership in response to COVID-19. American Psychologist. Advance online publication. http://dx.doi.org/10.1037/amp0000715

Thank you for the citation. We have included it, as well as three others, while mentioning Canada, Germany, and South Korea as three countries that appear to have coped with the initial wave of the pandemic more effectively than the U.S.

I generally like the additional commentary regarding the implications for the work, however, I wonder if something more could be said. I do not dispute that we should be looking to communicate accurate information and dispel myths, but this conclusion is fairly standard. Is it possible to say something more about how you could (perhaps) adapt your virtual behavior measures as an educational tool (bearing in mind the correlation with COVID-19 risk)? Also, maybe you could comment a bit more concretely about strategies that could be used to overcome the issues you outline (e.g. Hornesy and Fielding, 2017). I don’t think that there needs to be much added here, but a little more specificity around the guidance and suggestions would give the article more ‘bite’. Relatedly, adding in a few more references to back up you claims about how emotional appeals around vulnerability might be effective would be good.

Hornsey, M. J., & Fielding, K. S. (2017). Attitude roots and Jiu Jitsu persuasion: Understanding and overcoming the motivated rejection of science. American Psychologist, 72(5), 459-473. http://dx.doi.org/10.1037/a0040437

We are pleased to learn of your appreciation for the commentary we added regarding the implications of the work. We actually had cited the Hornsey & Fielding (2017) article in this additional commentary. We are reluctant to expand the discussion of strategies regarding scientific communication much, as that matter is well beyond the scope of the current research. However, we added a sentence highlighting the basic point offered by Hornsey & Fielding, as well as a few illustrative citations. Most importantly, we now conclude the paper with a brief paragraph considering the potential utility of our virtual behavior items as educational tools and efficacy measures. Thank you for the valuable suggestion.

*********

Once again, we wish to express our gratitude for the helpful comments offered by the reviewers in response to both the original and the revised manuscripts. We hope it is obvious that we have seriously considered each and every point that has been raised. We certainly believe that the various revisions have resulted in a stronger manuscript.

Sincerely,

Russell H. Fazio

Harold E. Burtt Chair in Psychology
